# Genome-Wide Identification of *TLP* Gene Family and Their Roles in *Carya cathayensis* Sarg in Response to *Botryosphaeria dothidea*

**DOI:** 10.3389/fpls.2022.849043

**Published:** 2022-04-01

**Authors:** Peipei Li, Yifan Xu, Ketao Wang, Wenlei Guo, Yujie Gu, Shiheng Lyu, Jianqin Huang, Haiping Lin, Chunying Huang, Zhen Xu, Yan Li

**Affiliations:** State Key Laboratory of Subtropical Silviculture, Zhejiang A&F University, Hangzhou, China

**Keywords:** *TLP* gene family, hickory, gene expressions, hormone, *B. dothidea*, subcellular localization

## Abstract

Hickory (*Carya cathayensis*) is a critical tree species of the genus *Carya* from the Juglandaceae family that contains nutrient-rich nuts. Due to large-scale soil degradation, the pests and diseases of hickory are becoming more and more serious. Thaumatin-like proteins (TLPs) are vital proteins involved in the complex defense process of plant pathogens. In this study, 40 *CcTLP* genes were identified genome-widely and phylogenetically grouped into three subfamilies. The sequence of *CcTLPs* had a conservative pattern, such as eight stable disulfide bonds, REDDD, and G-X-[GF]-X-C-X-T-[GA]-D-C-X(1,2)-G-X-(2,3)-C structure. In total, 57 *cis*-elements related to stress-responsive, light-responsive, phytohormone-responsive, and plant-responsive were discovered. Under salicylate (SA), methyl jasmonate (MeJA), and ethephon (ETH) treatments, the expressions of *CcTLP28, CcTLP29, CcTLP30, CcTLP31, CcTLP32, CcTLP33, CcTLP37, CcTLP38*, and *CcTLP39* had different patterns. This is an indication that most of the *TLP* genes were upregulated by SA and downregulated by MeJA. Notably, seven *TLP* genes were significantly upregulated under the *Botryosphaeria dothidea* inoculation, especially *CcTLP31*, with an over 20-fold change. Nine genes were shown by subcellular localization analysis to be located at the plasma membrane and cytoplasm. The knowledge of the disease-resistant function of the *CcTLP* family in hickory is promoted by these results. A foundation reference for the molecular breeding of this plant in the future is provided by our findings.

## Introduction

Plants are constantly attacked by various pathogens (e.g., bacteria, fungi, and viruses) in nature. In the long-term evolutionary process, plants and pathogens interact, adapt, and co-evolve. Plants have gradually established a series of complex defense mechanisms that coordinate well against the infection of pathogenic bacteria. Systemic-acquired resistance (SAR) is an essential defense mechanism induced by pathogenetic invasion (van Loon et al., 2006). Pathogenesis-related proteins (PRs) are induced in pathological or related situations intensely involved in the SAR process. So far, PRs have been identified and divided into 17 categories (PR1 to PR17) (van Loon et al., 2006; Dodds and Rathjen, 2010; Hamamouch et al., 2011). The PR-5 family, named as thaumatin-like proteins (TLPs), was first discovered in the fruit of *Thaumatococcus daniellii*, a shrub plant growing in the West African rainforest (Wel and Loeve, 1972). Afterward, *TLP* genes were explored in nematodes (Kitajima and Sato, 1999), insects (Brandazza et al., 2004), fungi (Grenier et al., 1999; Sakamoto et al., 2006), and multiple plants, such as *Arabidopsis thaliana*, *Oryza sativa*, *Populus trichocarpa*, *Manihot esculenta*, and *Cucumis melo* (Reimmann and Dudler, 1993; Abad et al., 1996; Midoro-Horiuti et al., 2000; van Loon et al., 2006; Liu et al., 2010b,^2020^; Petre et al., 2011; Irigoyen et al., 2020). A typical TLP protein sequence contains a highly conserved motif: G-X-[GF]-X-C-X-T-[GA]-D-C-X(1,2)-G-X-(2,3)-C (Jami et al., 2007; Tachi et al., 2009), a REDDD structure, and 5–8 disulfide linkages composed of 10 or 16 cysteine residues. These structures are thought to be associated with an antifungal function (Hu and Reddy, 1997). With the presence of these disulfide linkages, correct folding under unsuitable circumstances, such as extreme heat, low pH, and protease degradation, is allowed (Fierens et al., 2009). The N-terminal signal peptides of *TLP*s consist of 15–30 amino acids, with which the transportation from the ribosome to the extracellular fluid through the endoplasmic reticulum is ensured (Anžlovar and Dermastia, 2003). It was shown that the 3D structures of *TLP*s contained three domains, and domain I was located between domain II and III. There is a cleft structure between domains I and II, which is usually acidic and associated with pathogenic resistance (Rajam et al., 2007; Ghosh and Chakrabarti, 2008).

Members of the *TLP* family have been reported in recent studies to participate in multiple biological processes under biotic or abiotic stress (Fierens et al., 2009; Petre et al., 2011). For example, it has been found in several studies that TLPs increase the β-1,3-glucanase activity or are performed as xylanase inhibitors to destroy fungal cell walls (Jami et al., 2007; Martin et al., 2007). The resistance to rice sheath blight *Rhizocotonia solani* and sheath rot *Rocladittm oryzae*, is improved by the overexpression of *TLPs* in elite indica rice cultivars (Kalpana et al., 2006). Transgenic cassava with an overexpressing rice *TLP* gene significantly delayed anthracnose disease and enhanced fungal tolerance compared with wild types (Odeny Ojola et al., 2018). Moreover, an overexpression of the grape *TLP* gene in *A. thaliana* resulted in its more robust resistance to powdery mildew and *Pseudomonas syringae* pv. tomato *DC3000* compared with the wild type (Yan et al., 2017). In addition, it has been found that *BanTLP*s purified from banana extracts can serve as a defense against *Penicillium expansum* through the disturbance of the plasma membrane and cell wall disorganizing (Jiao et al., 2018). Recently, it has been shown that *GbTLPs* in *Gossypium barbadense* were significantly upregulated after *Verticillium dahlia* infection, suggesting a role for *TLP* in disease defense (Zhang et al., 2021). Furthermore, the tolerance of tobacco to salt, oxidative, and drought stress was enhanced by *AdTLPs* expression (Barthakur et al., 2001; Singh et al., 2013). Notably, it has been reported that SA and JA/ETH are involved in plant immunity. Resistance against biotrophic and hemi-biotrophic microbes is mediated by SA signaling, while JA and ethylene signaling participate in resistance against necrotrophs in plants (Gimenez-Ibanez et al., 2017).

Hickory (*Carya cathayensis*) is a commercially cultivated nut tree (Zhang and Xu, 2011), mainly distributed in China (Zhang and Xu, 2011). Its nut has a high nutritional value for human health, containing several nutrients, such as unsaturated fatty acids, flavonoids, dietary fibers, minerals, vitamins, and others (Huang et al., 2021, 2022; Li et al., 2022). With global climate change and soil over-utilization, soil degradation, biotic, and abiotic stresses are becoming more serious, resulting in the recent outbreak of a canker disease caused by *Botryosphaeria dothidea* (Zhang and Xu, 2011). This disease causes damage or death to hickory trees, bringing considerable losses in nut production and restricting its expanding cultivation. It has been revealed that *B. dothidea* causes infection symptoms on the trunk and branches, leading to tissue necrosis and disease spots around the infection sites, and disrupting the transportation of water and nutrients in the plants (Slippers et al., 2007). Therefore, we must explore the resistance genes in *C. cathayensis* against *B. dothidea*, which will contribute to the management of this disease.

In this study, we conducted the bioinformatics analyses, real-time fluorescence quantitative polymerase chain reaction (RT-qPCR), and *Agrobacterium*-mediated transient expression in tobacco for the genome-wide identification and analyzing the *TLP* family members in hickory (*C. cathayensis*). The objectives of this study were: (1) The identification, genomic location analysis, and protein characterization of the *CcTLP* genes, (2) to perform the multiple-alignment and phylogenetic analysis of the *CcTLPs* genes, (3) to analyze the gene structure, motif distribution, and *cis*-acting element of the *CcTLPs* genes, (4) to perform a differential expression profiling of the *TLP* genes under salicylate (SA), methyl jasmonate (MeJA), and ethephon (ETH) treatments, and infection by *B. dothidea*, and (5) to perform a subcellular localization assay of the *TLP* genes. With these studies, the disease resistance of the *CcTLP* family members in hickory will be better understood, and guidance for its molecular breeding improvement in the future will be provided.

## Materials and Methods

### Identification of Thaumatin-Like Protein Genes in Green Plants Genome

The whole genomic sequences of pecan (*Carya illinoinensis*), walnut (*Juglans regia*), and hickory (*C. cathyensis*) are obtained from the Portal of Juglandaceae (Guo et al., 2020). Genomic data of ginkgo (*Ginkgo biloba*), amborella (*Amborella trichopoda*), waterlily (*Nymphaea coloratar*), soybean (*Glycine max*), grape (*Vitis vinifera*), rice (*Oryza sativa*), and Phalaenopsis (*Phalaenopsis aphrodite*) were obtained from the National Center for Biotechnology Information (NCBI) genome database. The protein sequences of *A. thaliana* were derived from TAIR^1^.

A TLP keyword search of the NCBI for genes was conducted to find the coding sequence (CDS) of *TLP*, which were downloaded in FASTA format. The obtained *TLP* seed sequences were then searched against the previously downloaded whole-genome protein sequences using the BLASTX program (Camacho et al., 2009) (NCBI-BLAST 2.9.0 software, *E*-value of 10^–10^) to look for similar homologous protein sequences of the candidate *TLP* genes in the 10 species. The seed files of *TLP* domains (PF00314) were downloaded from the Pfam database.^2^ Candidate TLP protein sequences were further screened using the Hidden Markov Model algorithm of the HMMER software (Finn et al., 2011) and finally renamed as *CcTLP1*–*40*.

### Bioinformatic Analysis of Thaumatin-Like Protein Sequences

A portal, ExPASy,^3^ was used to identify and analyze the physicochemical properties of protein sequences (Gasteiger, 2003). Additionally, we predicted the location and transmembrane (TM) domains using Cell PLoc 2.0 (Chou and Shen, 2008) and TMHMM 2.0.^4^ The motif features of *TLP* sequences and signal peptides were analyzed with the MEME Suite and SignalP 4.0 (Petersen et al., 2011). Genome structure visualization was accomplished using the Gene Structure Display Server 2.0 online website (Hu et al., 2015) with a gff file. Promoter sequences were extracted using a pipeline of SAMtools and bedtools (Danecek et al., 2021). Then, the cis-acting elements were predicted using PlantCARE (Lescot, 2002).

MCSCAN software (Wang et al., 2012) was used to run the Pairwise Synteny Search program, with the whole-genome CDS of hickory and the gff file as input. The obtained anchor pairwise results were filtered with a 30-gene threshold for small fragment gene blocks. The identified *TLP* genes were searched in the final gene blocks.

MicroRNA (miRNA) is a class of non-coding single-stranded RNA molecules with a length of approximately 22 nucleotides that participate in the post-transcriptional gene expression regulation in plants and animals. The network profile between miRNAs and *CcTLP*s was further analyzed to better understand the miRNA regulation network of hickory *TLP* genes. The psRNATarget online server (Dai et al., 2018) was used to identify the miRNAs interacting with the hickory *TLP* genes. Then, the complex networks integrating miRNAs and *TLP* genes were visualized using Cytoscape (Shannon et al., 2003).

### Phylogenetic Analysis of the Thaumatin-Like Protein Family

Multiple sequence alignment of *TLP* genes was achieved using MAFFT software (Katoh, 2002) under local pair mode, and the maxiterate parameter was set to 1,000. Then, TrimAL software (Capella-Gutierrez et al., 2009) was utilized to remove the spurious sequences and poorly aligned regions. The final alignment result was removed with more than 20% gaps, a similarity score lower than 0.001, and minimum conservation of 60%. The phylogenetic tree was built using the maximum likelihood method using IQTREE software (Nguyen et al., 2015). The s parameter was used to estimate the optimal substitution model automatically during the calculation process, and the bootstrap value was set to 1,000. JTT + I + G4 and JTT + R7 were the best-fit substitution models for hickory and green plants trees sorted by Bayesian information criterion (BIC) scores. The generated tree file was transfigured with the Evolview3 website (Subramanian et al., 2019).

### Plant Materials and Hormone Treatments

In this experiment, the 6-month hickory seedlings growing in the Zhejiang Agriculture and Forestry University greenhouse (50 m, N30°23′, E119°72′) under a 16-h light/8-h dark photoperiod and at 22–25°C temperature were sprayed with 100 μM of SA, MeJA, and ETH until the first drop of liquid on the leaf surface. After treatments for 0, 2, 6, 12, 24, 48 h, 3–6 leaves from each plant were harvested, immediately frozen in liquid nitrogen, and stored at −80°C until use. This experiment was conducted with three independent biological replicates for each treatment.

### *Botryosphaeria dothidea* Culture and Its Inoculation on *Carya cathayensis*

The 2-year hickory seedlings were selected for *B. dothidea* inoculation in the Zhejiang Agriculture and Forestry University greenhouse (50 m, N30° 23′, E119° 72′). Before inoculation, *B. dothidea* was grown on potato dextrose agar (PDA) at 28°C in the dark. After 7 days, a 5-mm-diameter fungal agar plug was acquired using a punch and immediately placed on the surfaces of the wound site of the hickory stalk caused by the inoculating needle. In addition, a 5-mm-diameter empty PDA was used as control. The plug was removed after 2 days and the diameter of the disease areas was measured in six seedlings at 3, 4, 5, 6, 7, 9, 10, 11, 12, 14, and 16 days after inoculation (dpi) using rulers. The disease areas were collected at 0, 2, 7, and 16 dpi (Wang et al., 2020). All samples were immediately frozen in liquid nitrogen and then stored at −80°C until use. Each treatment contained three independent biological replicates.

### RNA Extraction, cDNA Synthesis, and Gene Expression Analysis

Total RNA was extracted using a Quick RNA Isolation Kit (Huayueyang, China). cDNA was synthesized using the PrimeScript™ 1st strand cDNA Synthesis Kit (Takara, Japan) according to the manufacturer’s instructions. The gene-specific primers of *TLPs* and SA-synthesis and signaling-related genes were designed using the online software Primer 3.^5^ All primers used in this study are listed in Supplementary Table 1. The quantitative reverse transcription (qRT-PCR) was conducted using the SYBR Green Master Mix reagent (Applied Biosystems) and CFX 96 Real-Time system (Applied Biosystems), according to the manufacturer’s instructions. *CcActin* was used as an internal standard for normalization. The reaction procedure was 40 cycles with 95°C for 10 s and 55°C for 30 s. Formula 2^–ΔΔCT^ was applied to calculate the relative expression. Each sample was conducted with three biological replicates and three technical replicates in the RT-qPCR experiment. The relative expression level of *CcTLP* genes was calculated by the standard curve and then normalized by the *CcActin* expression level. R-package pheatmap was utilized to plot the expression heatmap of *CcTLPs* with SA, MeJA, and ETH treatments for 0, 2, 6, 12, 24, and 48 h.

### Subcellular Localization Analysis

The full-length CDS of *CcTLP28, CcTLP29, CcTLP30, CcTLP31, CcTLP32, CcTLP33, CcTLP37, CcTLP38*, and *CcTLP39* were amplified with PCR. The gene-specific primers of the *TLPs* were designed using the Snapgene software and are listed in Supplementary Table 2. The PCR products were cloned into the binary vector 35s:GFP (modified from pCAMBIA 1300). The resulting plasmids with the correct sequence were introduced into *Agrobacterium tumefaciens* strains GV3101 and were cultured on the luria broth (LB) solid medium counting 50 μg/ml gentamicin (Geta), 50 μg/ml rifampicin (Rif), and 50 μg/ml kanamycins (Kana) at 28°C in the dark for 2 days. Then, a single colony was obtained, transformed into a liquid LB medium, and cultured at 28°C. After another 2 days, the cultures of *A. tumefa*ciens (OD600 = 0.5–0.6) were centrifuged at 5,000 rpm at room temperature for 10 min and re-suspended in MMA buffer (10 mM MES, 10 mM MgCl_2_, and 150 μM acetosyringone, pH = 5.6) to an OD600 of 1.0 and then incubated at room temperature in the darkness. After 2∼3 h, the suspension was injected into the 4-week-old tobacco (*Nicotiana benthamiana*) leaves. The plasma membrane marker (pm-rk) was used as a plasma membrane marker and was co-transformed with *CcTLPs* in tobacco (Nelson et al., 2007). After 2 days, green fluorescent protein (GFP) fluorescence was observed using laser confocal fluorescence microscopy (excitation: 488 nm; emission: 495–515 nm; LSM 800, Zeiss, Germany).

### Statistical Analysis

Statistical analyses were conducted using the one-way analysis of variance (ANOVA) procedure with SPSS (ANCOVA; SPSS26, SPSS Inc., Chicago, IL, United States). Significant differences among the groups were compared according to Duncan’s new multiple range test at *p* = 0.05.

## Results

### Identification and Genomic Location Analysis of the *CcTLP* Gene Family

After blasting and searching against the HMM seed model with whole-genome protein sequences, 40 *TLP* genes with typical thaumatin-like domains were found in *C. cathayensis* (Table 1). The number of *TLP* genes in 10 other green plants (*G. biloba*, *A. trichopoda*, *N. coloratar*, *O. sativa*, *P. aphrodite*, *A. thaliana*, *G. max*, *V. vinifera*, *J. regia*, and *C. illinoinensis*) are also listed in Table 1. Specifically, it has been shown that the amborella genome owns the least *TLP* genes. Compared with amborella, the number of *TLP* genes is almost two times in water lily belonging to the same Amborellales-Nymphaeales-Austrobaileyales (ANA) clade due to the Nymphaeaceae-specific whole-genome duplication (WGD) event (Zhang et al., 2020). Soybean and walnut genomes have the highest number of *TLP* genes, 62 and 66, respectively, almost three times than that of amborella, which may be attributed to that modern soybean was a diploid species from ancient tetraploid (Schmutz et al., 2010) and high quality of walnut genome (Marrano et al., 2020). From the information detected by the synteny analysis, four tandem duplications occurred in the distribution of *TLP* genes in hickory: *CcTLP07* and *CcTLP08* on the scaffold 18,053, *CcTLP15*, *CcTLP16*, and *CcTLP17* on the scaffold 25,681, *CcTLP21* and *CcTLP22* on the scaffold 28,723, *CcTLP29*, *CcTLP30*, *CcTLP31*, *CcTLP32*, and *CcTLP33* on the scaffold 54,619. Further synteny analysis with chromosome-level pecan genome showed these tandem duplicated *TLP*-located scaffolds originated from different chromosomes (Supplementary Figure 1). Five pairs of genes were revealed by gene synteny analysis to be duplicated from the WGD event, containing *CcTLP15*-*CcTLP22*, *CcTLP16*-*CcTLP22*, *CcTLP17*-*CcTLP21*, *CcTLP18*-*CcTLP25*, and *CcTLP31*-*CcTLP38*, which implied a potential influence of WGD event on *CcTLP* gene expansion.

**TABLE 1:** Identified *TLP* gene count after the program of BLASTx and HMM Search in 11 plants.

Species	Gene count	
	BLASTx	HMM Search
*Ginkgo biloba*	69	37
*Amborella trichopoda*	50	19
*Nymphaea coloratar*	70	33
*Oryza sativa*	69	37
*Phalaenopsis aphrodite*	48	22
*Arabidopsis thaliana*	65	32
*Glycine max*	84	62
*Vitis vinifera*	64	33
*Juglans regia*	91	66
*Carya illinoinensis*	74	43
*Carya cathayensis*	73	40

### Analysis of the Physical and Chemical Characteristics of *CcTLP* Proteins

Protein characteristics of hickory *TLP* gene family were further analyzed and presented in Table 2, such as Instability index (II), Aliphatic index (AI), Grand average of hydropathicity (GRAVY), and isoelectric point (pI). The II ranges from 26.65 (*CcTLP29*) to 73.31 (*CcTLP08*), and the AI values were between 52.1 (*CcTLP35*) and 83.67 (*CcTLP27*). According to GRAVY values, 35% (14 out of 40) of *TLP* genes code as hydrophobic, while others code as hydrophilic proteins. The pI values of 10 out of 40 *CcTLP* proteins reached values higher than seven, while the remaining ones reached values lower than seven. The vast majority of *TLP* genes were shown by sublocalization predication to be distributed in the cytoplasm. The exceptions were *CcTLP14* and *CcTLP18*, located in the cell membrane/cytoplasm and vacuole, respectively. Finally, most of the genes contained transmembrane domains (Table 2). Notably, it was also found that some of these TLPs had N-signal peptides and transmembrane domains (TM) (Table 2).

**TABLE 2:** Protein features of *TLP* gene family in hickory.

Gene ID	Protein characteristics						
	II	AI	GRAVY	pI	Localization	Numbers of TM	Signal peptides
*CcTLP01*	42.71	75.25	0.055	8.62	Cytoplasm	2	Yes
*CcTLP02*	48.43	61.38	–0.194	4.5	Cytoplasm	0	Yes
*CcTLP03*	41.3	66.21	–0.044	4.87	Cytoplasm	0	Yes
*CcTLP04*	41.87	60	–0.028	4.51	Cytoplasm	0	Yes
*CcTLP05*	43.66	59.92	–0.062	4.43	Cytoplasm	0	Yes
*CcTLP06*	48.8	61.74	–0.074	4.62	Cytoplasm	0	Yes
*CcTLP07*	57.95	67.45	0.048	4.13	Cytoplasm	0	No
*CcTLP08*	73.31	59.87	–0.315	7.97	Cytoplasm	0	No
*CcTLP09*	57.29	68.98	0.04	4.45	Cytoplasm	0	Yes
*CcTLP10*	48.01	55.34	–0.147	4.43	Cytoplasm	0	No
*CcTLP11*	41.17	57.85	–0.141	4.62	Cytoplasm	0	Yes
*CcTLP12*	41.66	75.51	0.118	4.71	Cytoplasm	1	Yes
*CcTLP13*	38.67	72.13	–0.046	8.62	Cytoplasm	1	Yes
*CcTLP14*	46.05	58.86	–0.087	4.32	Cell membrane/cytoplasm	1	Yes
*CcTLP15*	43.71	62.55	–0.086	4.88	Cytoplasm	2	Yes
*CcTLP16*	43.71	62.55	–0.086	4.88	Cytoplasm	2	Yes
*CcTLP17*	32.49	73.21	0.05	5.53	Cytoplasm	1	Yes
*CcTLP18*	49.34	74.63	–0.013	8.27	Cytoplasm	1	Yes
*CcTLP19*	37.03	57.69	–0.202	7.37	Vacuole	1	Yes
*CcTLP20*	42.1	82.41	0.193	8.36	Cytoplasm	0	Yes
*CcTLP21*	39.15	67.26	–0.035	4.46	Cytoplasm	1	Yes
*CcTLP22*	42.61	58.59	–0.058	4.58	Cytoplasm	1	Yes
*CcTLP23*	42.96	59.84	–0.14	5.44	Cytoplasm	0	Yes
*CcTLP24*	51.32	67.96	–0.045	7.23	Cytoplasm	1	Yes
*CcTLP25*	47.85	71.93	–0.031	7.39	Cytoplasm	1	Yes
*CcTLP26*	32.65	70.32	0.02	6.83	Cytoplasm	0	Yes
*CcTLP27*	44.87	83.67	–0.077	5.17	Cytoplasm	1	Yes
*CcTLP28*	47.45	79.05	0.023	8.26	Cytoplasm	0	Yes
*CcTLP29*	26.65	73.16	0.072	4.35	Cytoplasm	1	Yes
*CcTLP30*	32.15	75.88	0.015	4.29	Cytoplasm	0	Yes
*CcTLP31*	27.5	64.78	–0.04	4.27	Cytoplasm	0	No
*CcTLP32*	30.19	74.07	0.034	4.24	Cytoplasm	0	Yes
*CcTLP33*	27.78	74.21	0.21	4.3	Cytoplasm	1	Yes
*CcTLP34*	41.04	68.96	0.211	4.45	Cytoplasm	2	Yes
*CcTLP35*	43.26	52.1	–0.328	5.34	Cytoplasm	0	No
*CcTLP36*	36.66	78.63	0.1	8.05	Cytoplasm	1	Yes
*CcTLP37*	44.79	67.93	–0.075	8.44	Cytoplasm	0	Yes
*CcTLP38*	36.51	61.77	0.034	4.79	Cytoplasm	0	Yes
*CcTLP39*	41.46	68.27	0.044	5.2	Cytoplasm	1	Yes
*CcTLP40*	48.77	62.28	–0.301	4.59	Cytoplasm	0	No

### Multiple-Alignment Analysis of Hickory Thaumatin-Like Proteins

**FIGURE 1:**
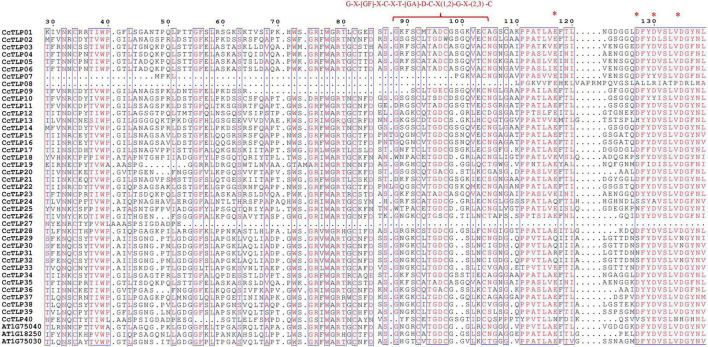
Multiple alignment sequence of 43 conserved thaumatin-like protein (*TLP*) regions. Bracket indicated the G-X-[GF]-X-C-X-T-[GA]-D-C-X(1,2)-G-X-(2,3)-C region, asterisk signs represented the highly conversed REDDD structure.

The multi-sequence alignment was further performed and was shown in Figure 1. Most hickory TLP peptide sequences (35 out of 40) contain 16 cysteine residues and form eight stable disulfide bonds. However, *CcTLP07*, *CcTLP09*, and *CcTLP27* lost five cysteines that only allowed them to encode type S (Small) TLPs with anti-fungal activity (Liu et al., 2010a). Moreover, it was observed that the *CcTLP02* and *CcTLP09* sequences were missing one and three amino acids, respectively. The REDDD structure from several genes has undergone varying degrees of mutation and deletion. For instance, *CcTLP27* and *CcTLP40* completely lost their REDDD structures, which may lead to an inability to maintain proper topologies (Liu et al., 2012). It was found that *CcTLP24*, *CcTLP08*, *CcTLP29*, *CcTLP30*, and *CcTLP32* suffered 2–3 site mutations that may have affected their anti-microbial function. Notably, most of the hickory *TLP* sequences reversed the core domain sequence G-X-[GF]-X-C-X-T-[GA]-D-C-X(1,2)-G-X-(2,3)-C. Still, *CcTLP27* missed part of the sequence: G-X-[GF]-X-C-X-T.

### Phylogenetic Analysis of the Thaumatin-Like Proteins of Hickory

**FIGURE 2:**
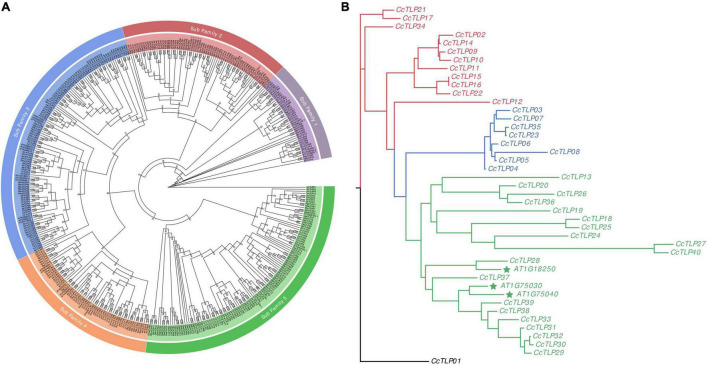
Hickory phylogenetic tree constructed with 424 plant *TLP* genes **(A)** and full-length peptides sequences of 40 and three *TLPs* from hickory and three *Arabidopsis thaliana*, respectively **(B).** The phylogenetic tree of 424 plant *TLP* genes was plotted with the maximum-likelihood estimation, JTT + R7 substitution model, and 1,000 bootstrap times. The phylogenetic tree was divided into five clades: Purple, dark red, blue, orange, and green. The 43 *TLP* peptides sequences were aligned using the MAFFT software. Then, the phylogenetic tree was inferred with IQTree with the parameters of the JTT + I + G4 substitution model and 1,000 times bootstrap. The maximum-likelihood tree could be divided into three groups, presented by red, green, and blue lines.

A total of 424 *TLP* genes were identified in 11 green plants to analyze the evolutionary relationship of *TLP* genes among green plants. The phylogenetic tree was distinctly divided into five subfamilies (Figure 2A). The green clade had the largest number of *TLP* genes (115, including 18 hickory *TLP* genes). Notably, this clade contains 37 gingko *TLP* genes and basal amborella *TLP* genes. This finding is an indication that the clade represents a complete evolutionary history. Blue, red, and purple clades had 117 (27.6%), 70 (16.5%), and 42 (9.91%) *TLP* genes, respectively. The red clade had 33 Juglandaceae species, consisting of 47.1% of the 70 *TLP* genes. This finding is a suggestion that the Juglandaceae-specific *TLP* gene family expansion was within the red clade.

A maximum-likelihood tree was constructed using 43 full-length *TLP* sequences (40 from hickory and three from *Arabidopsis thaliana*) to explore the further phylogenetic relationship among *TLP* genes in hickory (Figure 2B). The maximum-likelihood tree can be divided into three groups: group I (red line), group II (blue line), and group III (green line). The richest group III contained 22 *TLP* genes, including 19 *CcTLP* genes and three *Arabidopsis TLP* genes, which have been significantly studied. Within group III, *CcTLP27* and *CcTLP40* owned the longest clades, 2.6292 and 2.5801, respectively, indicating that these two genes were highly diverged. This result was consistent with the loss of REDDD in these two genes. The gene count in groups I and II was 12 and 8, and gene branch lengths in these two groups were nearly the same apart from *CcTLP08*. These results are suggestions that the genetic variation within group I and group II was low, and *TLP* gene functions were potentially similar.

### Gene Structure and Motif Distribution of Thaumatin-Like Protein Genes

**FIGURE 3:**
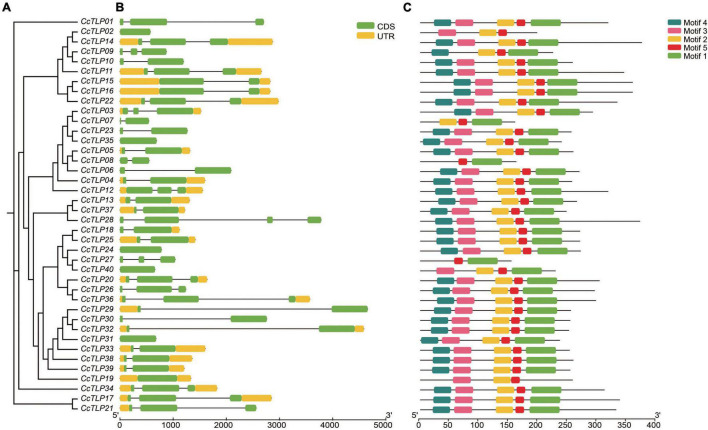
Rectangular phylogenetic tree, gene structure, and motif distribution of *CcTLP* genes. **(A)** The phylogenetic tree was inferred based on 40 hickory *TLP* peptides sequences. **(B)** Gene structure of Cc*TLP* genes including 5′ and 3′-UTR (yellow bar), exon (green bar), and intron (black line). **(C)** Motif patterns in 40 *CcTLP* genes.

Differences in the type and arrangement order of exons and introns could impact gene function. The gene structure of *CcTLPs* was analyzed and is shown in Figure 3B. Six *CcTLP* genes (*CcTLP02*, *CcTLP35*, *CcTLP24*, *CcTLP40*, *CcTLP19*, and *CcTLP31*) maintained only one exon, with no intron element within the genes. In the remaining, most of the genes (20 out of 35) contained two exons and one intron elements, 14 out of 35 genes had three exons and two introns, and remarkably *CcTLP28* comprised four exons. Peptide sequence regions that have a significant impact on protein function or structure should be more conservative, which are called motifs. According to the motif analysis results, five motif patterns were found within 40 *CcTLP* genes (Figure 3C). Most of the *CcTLP* genes (82.5%) maintained the complete five motif patterns, while *CcTLP09* and *CcTLP40* lost one motif pattern, *CcTLP02*, *CcTLP07*, and *CcTLP19* lost two motifs, and *CcTLP08* and *CcTLP27* only maintained two unbroken motifs.

### *Cis*-Acting Elements Analysis of the Thaumatin-Like Protein Gene

**FIGURE 4:**
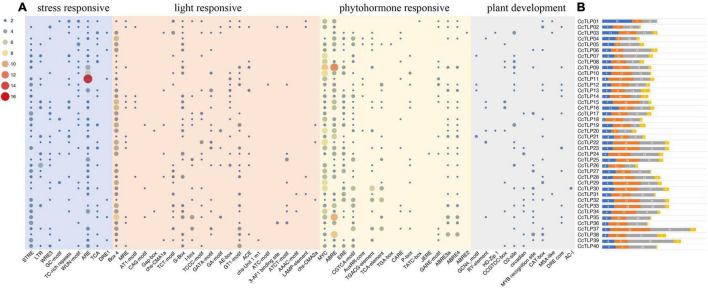
The predicted *Cis*-acting elements distribution pattern in 40 *CcTLP* gene promoter regions. **(A)** These *cis*-acting elements were classified into four groups: stress-responsive, light-responsive, phytohormone-responsive, and plant-responsive, as shown in the heatmap. **(B)** The total count of these four categories is displayed in the bar plot. Blue: stress-responsive. Orange: light-responsive. Gray: Phytohormone-responsive. Yellow: Plant-responsive.

*Cis*-acting elements are important short regions in the promoter sequence recognized by specific transcriptional factors, thereby regulating the activity of the promoter and the targeted gene expression. A batch of *cis*-acting elements was found by predicting the 2 kb *CcTLP* promoter sequences, such as light-responsive elements, hormonal regulation elements, and stress-related elements. Then, 65 emphasis elements related to stress-responsive, light-responsive, phytohormone-responsive, and plant-responsive were selected for classifying and accounting (Figure 4). Remarkably, the *CcTLP11* promoter sequence contained 16 ARE elements involved in the antioxidative response. The *CcTLP09* promoter sequence covered 12 ABRE elements, and 10 MYC elements participated in the phytohormone regulation process. The *CcTLP* genes were revealed by these results to be potentially involved in various life activity regulations, such as the antipathogen process, abiotic stress, and plant development.

### miRNA Prediction and the Interaction With Thaumatin-Like Protein Genes

**FIGURE 5:**
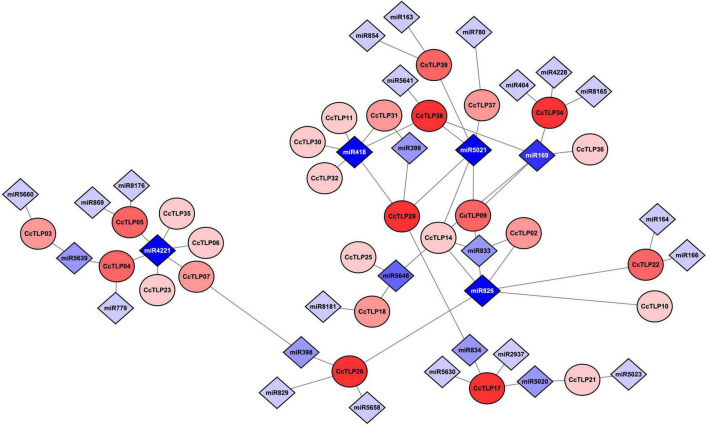
Interaction network diagram between 29 *CcTLP* genes and 31 micro RNAs (miRNAs). Circle elements represented *CcTLP* genes and rhombus elements represented miRNA interacting with *CcTLP* genes. The level of color depth indicated the co-regulation ability.

In Figure 5, thirty-one miRNAs and 20 *CcTLP*s were predicted to be involved in the post-transcriptional process. Most of the *CcTLPs* (16 out of 29) were connected with one or two miRNAs. In contrast, *CcTLP17*, *CcTLP26*, *CcTLP29*, *CcTLP34*, and *CcTLP38* were the most highly active genes to be influenced by four miRNAs. In addition, 24 out of 31 miRNAs targeted one or two *TLP* genes. Notably, miRNA4221 and miRNA5021 interacted with five *TLP* genes, suggesting that they had extensive roles in regulating the expression of *TLP* genes.

### Differential Expression Profiling of *CcTLP* Genes Under Different Plant Hormone Treatments

**FIGURE 6:**
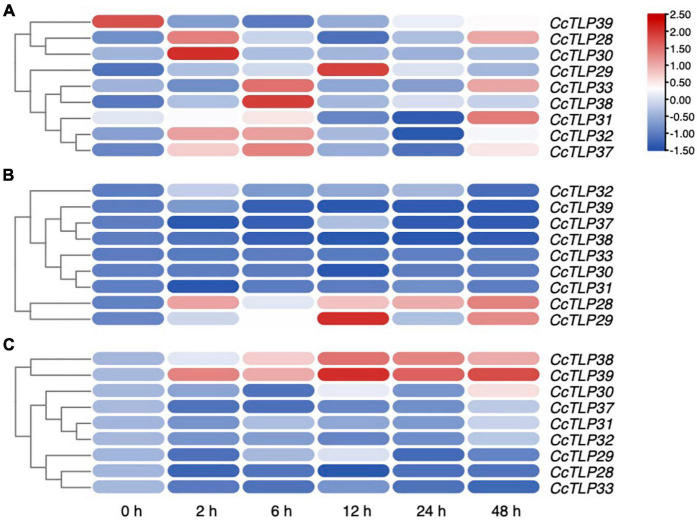
The expression patterns of nine *CcTLP* genes after plant hormones treatments. The relative expression of nine *CcTLP* genes after treatments with SA, MeJA, and ETH for 0, 2, 6,12, 24, and 48 h were calculated using quantitative real-time polymerase chain reaction (qRT-PCR) analysis. The expression level was represented by the mean relative expression value. The relative expression level of *CcTLP* genes was calculated by the standard curve and then normalized by the *CcActin* expression level. R-package pheatmap were utilized to plot the expression heatmap of *CcTLPs* after treatments with salicylate (SA), methyl jasmonate (MeJA), and ethephon (ETH) for 0, 2, 6, 12, 24, and 48 h. Color bar in the diagram showed the range of normalized signal intensities.

The SA and JA/ETH defense pathways are antagonistic, reflecting that enhanced resistance against biotrophs is often correlated with strengthened susceptibility to necrotrophs and vice versa. We selected nine *CcTLP* genes from the same clade with *AT1G75040*, *AT1G75030*, and *AT1G18250* to quantify their expression levels in leaves after treatments with SA, MeJA, and ETH using qRT-PCR and to investigate the role of *CcTLP* genes, as demonstrated in Figure 6. It was shown that these most genes were all upregulated under SA treatment, especially *CcTLP28*, *CcTLP29*, *CcTLP30*, *CcTLP33*, and *CcTLP38*. However, *CcTLP37, CcTLP38*, and *CcTLP39* were downregulated at all-time points under JA treatment. At the same time, *CcTLP28* and *CcTLP29* were significantly upregulated during all periods. Under ETH treatment, only two genes (*CcTLP38* and *CcTLP39*) were found to be significantly upregulated. *CcTLP29* and *CcTLP30* had various changes in expression levels, being upregulated at some points and downregulated at others. These data are indications that *CcTLP* genes had different sensitivities to exogenous SA, JA, and ETH applications. It was suggested that the sequence of the *CcTLP* genes shared a high identity. Nonetheless, they had different expression patterns in response to the same hormone.

### Different Expression Patterns of *CcTLP* Genes in Response to *Botryosphaeria dothidea* Infection

**FIGURE 7:**
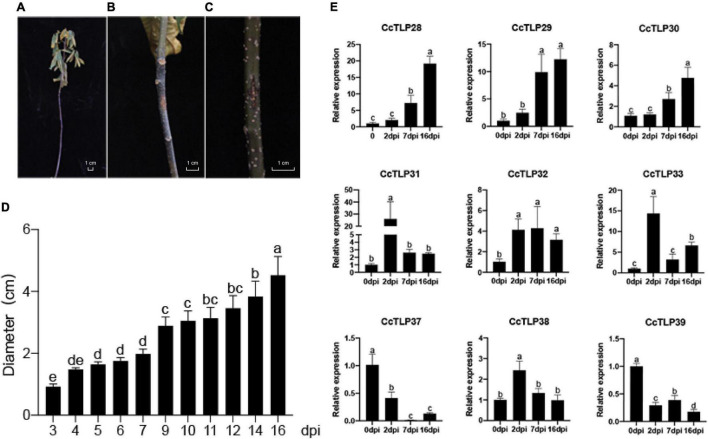
The phenotype and expression analysis of *CcTLP* genes in response to *Botryosphaeria dothidea* infection. **(A)** Plant phenotype after 14 days after inoculation (dpi). The leaves were dried and curly. **(B,C)** The disease areas of wound sites were applied to agar containing *B. dothidea* mycelia and empty 0.5-cm-diameter agar, respectively, after 14 dpi. **(D)** Analysis of the diameter length of disease areas at 3, 4, 5, 6, 7, 9, 10, 11, 12, 14, and 16 dpi. This is represented by the mean ± SE. Error bars were obtained from three technical repeats. **(E)** An expression analysis of *CcTLP* family genes under *B. dothidea* infection. Samples were collected at 0, 2, 7, and 16 days after treatment. Each bar is the mean ± SE from three technical repeats. Different letters in the error bar showed significant differences between the sample groups as determined using ANOVA (Duncan’s test, *p* < 0.05).

We further analyzed the changes in the previous nine *CcTLP* gene expressions to understand the function of *TLP* genes under infection conditions by *B. dothidea*. As shown in Figures 7A–C, *B. dothidea* caused dried and curly leaves, and the plant was led to death. When agar-containing mycelia was applied to the wound site, it became black, and the area enlarged was significantly different from control after 14 dpi. The diameters areas of the disease were broader after 3, 7, and 16 days of treatment (Figure 7D). Three *CcTLP* genes (*CcTLP28*, *CcTLP29*, and *CcTLP30*) were gradually upregulated with infection time. *CcTLP31, CcTLP32, CcTLP33, and CcTLP38* could also be upregulated, and their expression levels were the highest after 2 dpi. Notably, *CcTLP31* had an over 20-fold expression change. Based on these results, it is speculated that these genes were positively correlated with hickory resistance to *B. dothidea* (Figure 7E). In contrast, *CcTLP37* and *CcTLP39* were downregulated at the indicated time points compared with control (Figure 7E). This result is a suggestion that these *CcTLP* genes are negatively associated with hickory resistance to *B. dothidea.*

### Subcellular Localization of *CcTLPs*

**FIGURE 8:**
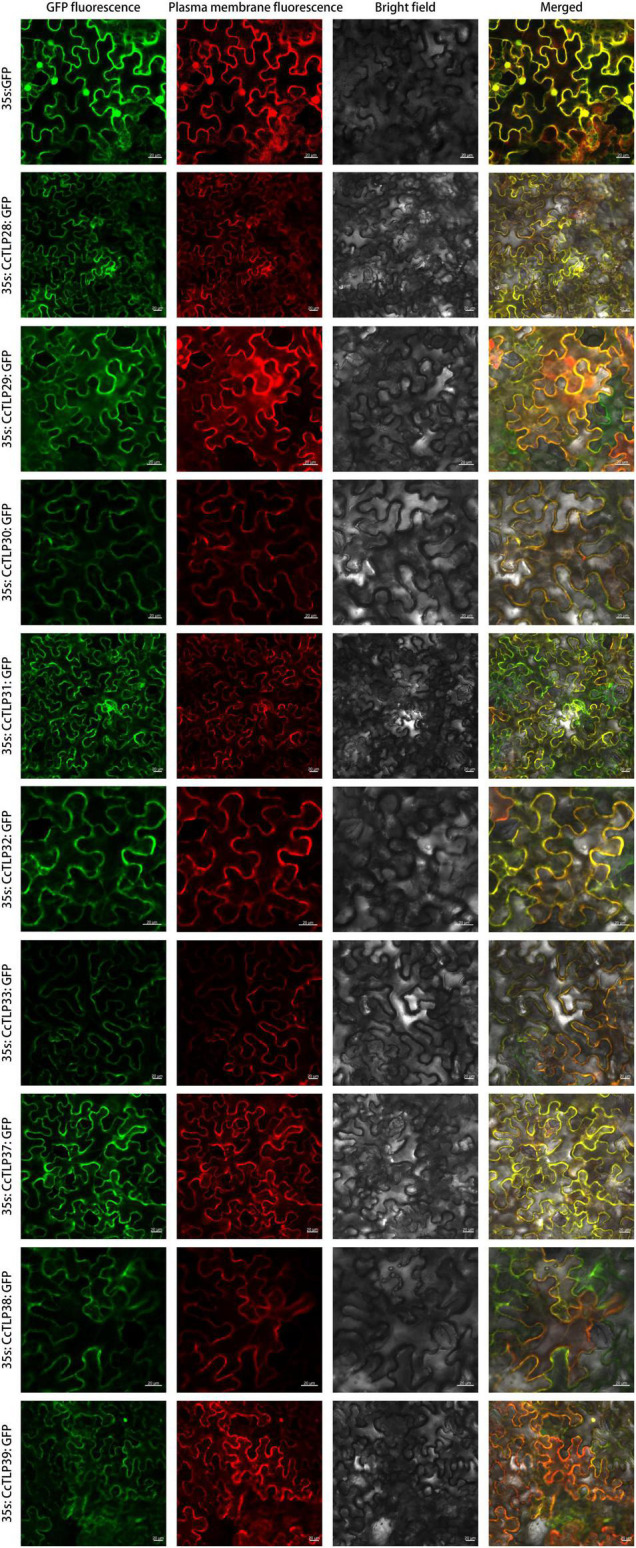
The subcellular localization analysis of the *CcTLP28, CcTLP29, CcTLP30, CcTLP31, CcTLP32, CcTLP34, CcTLP37, CcTLP38*, and *CcTLP39* in *Nicotiana. benthamiana*. The transient expression of the *35S::CcTLP-EGFP* fusing construct and the *35S::EGFP* construct in *N. benthamiana*. Green fluorescent protein (GFP) fluorescence was observed 3 dpi using a confocal microscope. Scale bars: 20 μm.

It has been reported that TLP proteins have antifungal functions. Subcellular localization analysis was further conducted to explore where they function. The GFP protein alone was present in the plasma membrane, cytoplasm, and nuclei, while the fluorescent signals of most CcTLPs-GFP were all found in the cytoplasm and plasma membrane (overlapped perfectly with the red fluorescence of the plasma membrane marker), as shown in Figure 8.

## Discussion

Hickory is a critical economic tree species from the Juglandaceae family. Hickory nuts have several superior nutritional qualities, such as high quantities of mono-unsaturated fatty acids, thiamine, and dietary fibers, which are beneficial for human health (Miraliakbari and Shahidi, 2008; Bolling et al., 2011). Although these benefits may promote the cultivation and commercial exploitation of hickory, its planting area is still restricted to limited areas, such as Anhui, Hangzhou, Guizhou, and Hunan provinces in China. This stress situation primarily results from its weak resistance to the environment and biotic stress (Yang et al., 2015; Grauke et al., 2016). Therefore, it is vital to understand the genetic basis of hickory and exhume resistant gene resources to abiotic and pathogen threats.

The *TLP* family members have essential functions in plant development and response to adversity stresses (Singh et al., 2013). In this study, 40 *TLP* family member genes were identified from the hickory genome using a pipeline of bioinformatics approaches, with more members than amborella (19), water lily (33), *Arabidopsis* (32), rice (37), ginkgo (37), *P. aphrodite* (22), grape (33), but fewer than soybean (62), walnut (66), and pecan (43). The widely available *TLP* genes have a vital role in plants. It has been shown in previous studies that plant *TLP* gene families are formed by the evolutionary amplification of 10 common ancestral genes before the divergence of monocotyledons and dicots. The diversity of *TLP* genes in terrestrial plants is significantly better than that in animals and fungi (Liu et al., 2010a). During the long way of the evolution of the *TLP* gene family, WGD is thought to be an essential driver of *TLP* gene expansion and a significant source of functional evolution. In our research, we found that four tandem duplicate events appeared in the *TLP* family, accounting for 30%. It was shown by gene synteny analysis that five pairs of genes emerged from the WGD event. The expansion of the *CcTLP* family in hickory could jointly be explained by these results (Figure 2).

Members of the same branch were revealed by gene structure and conserved motif analyses to have similar gene structures. Most had the same number of conserved motifs and distribution patterns, implying some conserved function among *CcTLP* members (Figure 3). For instance, most of the *CcTLPs* contained specific receptor binding sites for antifungal activity: G-X-[GF]-X-C-X-T-[GA]-D-C-X(1,2)-G-X-(2,3)-C and REDDD structure (Figure 1). A cis-acting element in the promoter interacts with specific transcription factors to form a transcription initiation complex that initiates gene-specific expression (Figure 4). *CcTLPs* contain many hormonal regulation elements, such as TGACG-motif involved in MeJA responsiveness and a TCA-element involved in SA responsiveness. Phytohormones, especially SA, JA, and ETH, function as key signaling molecules in the plant defense response under pathogen attack (Reymond and Farmer, 1998; Pieterse and van Loon, 1999; Thomma et al., 2001). It was shown by the results that the expression of *CcTLP* family genes could be influenced by SA, JA, and ETH. Especially under SA treatment, *CcTPL28*, *CcTPL30*, and *CcTPL32* reached the highest expression after 2 h (Figure 6). The *VqTLP29* transgenic lines have been revealed in previous studies to have improved resistance to powdery mildew and *Pst* DC3000 (Yan et al., 2017). In addition, the *TLP* transgenic poplars had an enhanced resistance against spot diseases (Sun et al., 2020). We accessed the expression of the *CcTLP* genes in hickory seedlings infected with *B. dothidea* to verify their roles in signaling pathways related to pathogen-induced stress. Similarly, it was found that the infection could significantly regulate seven *CcTLP* genes, except for *CcTLP37* and *CcTLP39* (Figure 7). Moreover, the expression of *CcTLP31*, *CcTLP33*, and *CcTLP38* peaked under the *B. dothidea* infection after 2 days. Additionally, the expression of SA synthetic-related genes phenylalanine ammonia lyase (*PAL*) and the non-expressor of pathogenesis-related genes 1 (*NPR1*) were observed to be increased, indicating the possible increasement of SA content after infection (Supplementary Figure 3). Consequently, we could hypothesize how *CcTLP* genes participated in disease resistance. Under *B. dothidea* infection, there is an increase of SA endogenous levels. Then, transcription factors combine on the SA responsive elements, causing the upregulation of many *TLP* genes against pathogen attacks.

For the localization of TLP proteins, *AdTLP* and *TaPR5* were identified as extracellular proteins (Wang et al., 2010; Singh et al., 2013). Other *TLPs*, *RlemTLP*, and *CsTLP1*, being predicted as extracellular, were found to be located in both the periphery of the plasma membrane and cytoplasm and also had an antifungal function (Garcia-Casado et al., 2000; Kim et al., 2009). The localization analysis of nine recombinant CcTLPs showed cytoplasm location (Figure 8), consistent with previous studies and location predictions (Table 2). Notably, it was observed that the fluorescent signals of most CcTLPs-GFP overlapped perfectly with the red fluorescence of the marker, suggesting that they were also present in the plasma membrane, which was different from the prediction. Therefore, the antifungal function of CcTLPs in hickory was inferred by us because of these results. Some of these TLPs contained N-signal peptides and transmembrane domains, which guided mature protein outward to participate in the defense response (Li et al., 2020). Therefore, it is likely that *CcTLPs* also have an antifungal function and are related to some cytoplasmic organs in cells.

## Conclusion

Thaumatin-like proteins are indispensable parts of the plant immune system that accumulate rapidly to a high content under biotic stress. In this study, 40 hickory *TLP* genes were identified and classified by phylogenetic relationship, gene structure, and motif distribution. The significant structure of eight stable disulfide bonds, REDDD, G-X-[GF]-X-C-X-T-[GA]-D-C-X(1,2)-G-X-(2,3)-C were discovered and signed. Fifty-seven *cis*-elements within 40 *CcTLP* genes related with stress, light, phytohormone, and plant responses were discovered. Among these genes, nine *CcTLP* genes distributed in the same cluster as *AtTLP* can be regulated by plant hormones, especially salicylic acid (SA). It was shown by qRT-PCR that the expression of seven *CcTLP* genes was significantly induced among the nine genes under *B. dothidea* inoculation. The expression of *CcTLP38*, *CcTLP32*, *CcTLP33, CcTLP30*, and *CcTLP31* was upregulated considerably with the treatments with SA and *B. dothidea* infection. The resistance against biotrophic and hemi-biotrophic microbes is mediated by SA-signaling. Therefore, when fungi attack plants, the endogenous level of SA may increase, causing the upregulation of the *TLP* gene secreted to the extracellular to achieve an antifungal effect. Using an assay of *N. benthamiana*, the CcTLP protein was indicated by the leave cell to be located in the plasma membrane and cytoplasm. The disease-resistant function in *CcTLP* family was explored in the results, providing helpful information for aiding the Chinese hickory molecular breeding improvement process.

## Data Availability Statement

The original contributions presented in the study are included in the article/Supplementary Material, further inquiries can be directed to the corresponding author/s.

## Author Contributions

PL, YL, and JH conceived and designed this study. WG and PL analyzed the data. PL, YX, YG, and SL performed the experiments. PL and WG wrote the manuscript. YL, JH, and KW edited and reviewed the writing. HL and YG offered resources. All authors have read and approved this manuscript.

## Conflict of Interest

The authors declare that the research was conducted in the absence of any commercial or financial relationships that could be construed as a potential conflict of interest.

## Publisher’s Note

All claims expressed in this article are solely those of the authors and do not necessarily represent those of their affiliated organizations, or those of the publisher, the editors and the reviewers. Any product that may be evaluated in this article, or claim that may be made by its manufacturer, is not guaranteed or endorsed by the publisher.

## Footnotes

1
https://www.arabidopsis.org/

2
http://pfam.xfam.org/

3
https://web.expasy.org/protparam/

4
https://services.healthtech.dtu.dk/service.php?TMHMM-2.0

5
https://primer3.ut.ee/
