# Supplementary material for: Genome-Wide Identification of TLP Gene Family and Their Roles in Carya cathayensis Sarg in Response to Botryosphaeria dothidea
Source: Front Plant Sci. 2022 Apr 1;13:849043. doi: 10.3389/fpls.2022.849043 (PMC9010463; doi:10.3389/fpls.2022.849043)
Supplement: Supplementary file 1 [file Presentation_1.pptx]

## Slide 1
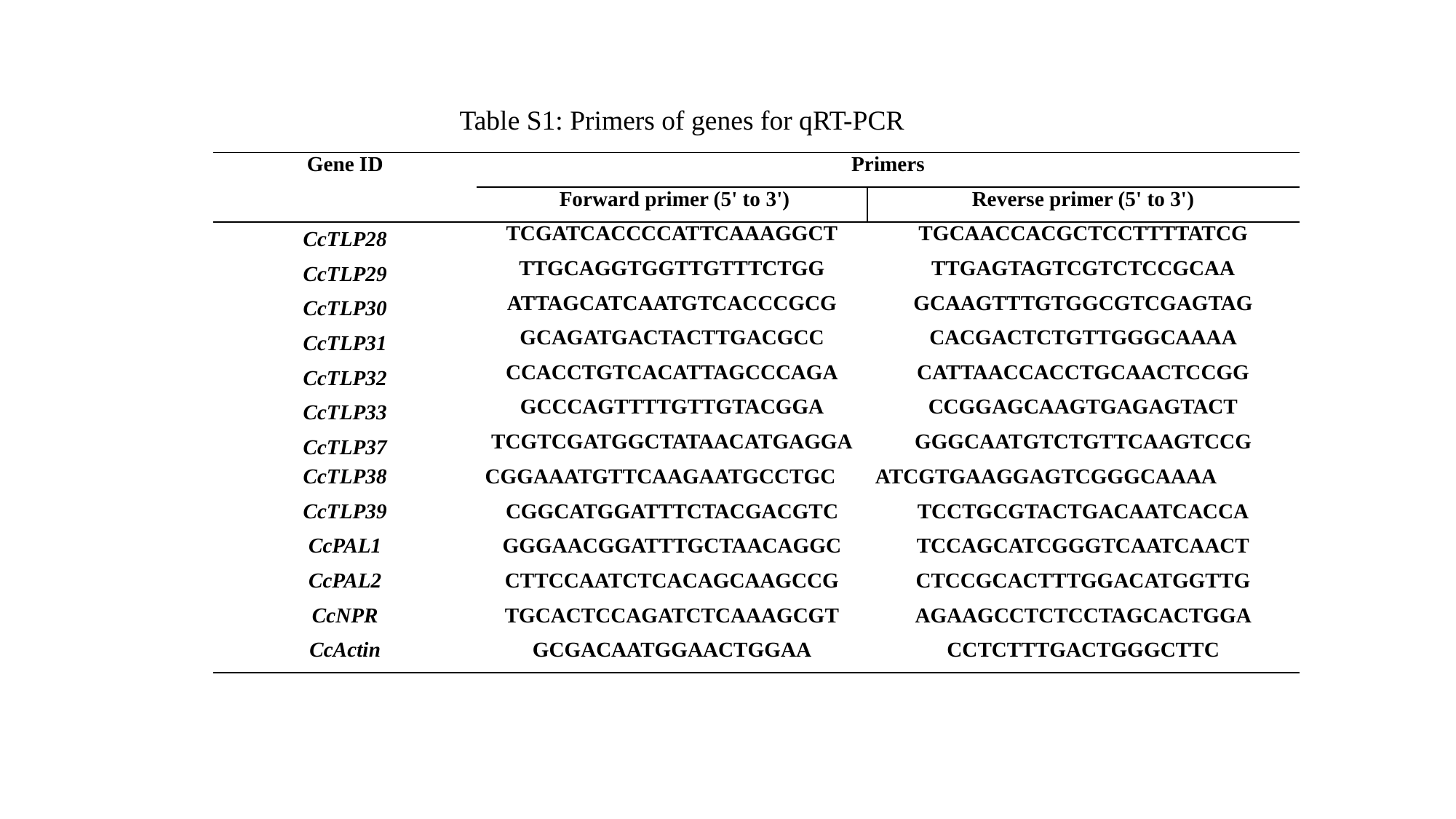

Table S1: Primers of genes for qRT-PCR
| Gene ID | Primers | |
| --- | --- | --- |
| | Forward primer (5' to 3') | Reverse primer (5' to 3') |
| CcTLP28 | TCGATCACCCCATTCAAAGGCT | TGCAACCACGCTCCTTTTATCG |
| CcTLP29 | TTGCAGGTGGTTGTTTCTGG | TTGAGTAGTCGTCTCCGCAA |
| CcTLP30 | ATTAGCATCAATGTCACCCGCG | GCAAGTTTGTGGCGTCGAGTAG |
| CcTLP31 | GCAGATGACTACTTGACGCC | CACGACTCTGTTGGGCAAAA |
| CcTLP32 | CCACCTGTCACATTAGCCCAGA | CATTAACCACCTGCAACTCCGG |
| CcTLP33 | GCCCAGTTTTGTTGTACGGA | CCGGAGCAAGTGAGAGTACT |
| CcTLP37 | TCGTCGATGGCTATAACATGAGGA | GGGCAATGTCTGTTCAAGTCCG |
| CcTLP38 | CGGAAATGTTCAAGAATGCCTGC | ATCGTGAAGGAGTCGGGCAAAA |
| CcTLP39 | CGGCATGGATTTCTACGACGTC | TCCTGCGTACTGACAATCACCA |
| CcPAL1 | GGGAACGGATTTGCTAACAGGC | TCCAGCATCGGGTCAATCAACT |
| CcPAL2 | CTTCCAATCTCACAGCAAGCCG | CTCCGCACTTTGGACATGGTTG |
| CcNPR | TGCACTCCAGATCTCAAAGCGT | AGAAGCCTCTCCTAGCACTGGA |
| CcActin | GCGACAATGGAACTGGAA | CCTCTTTGACTGGGCTTC |

## Slide 2
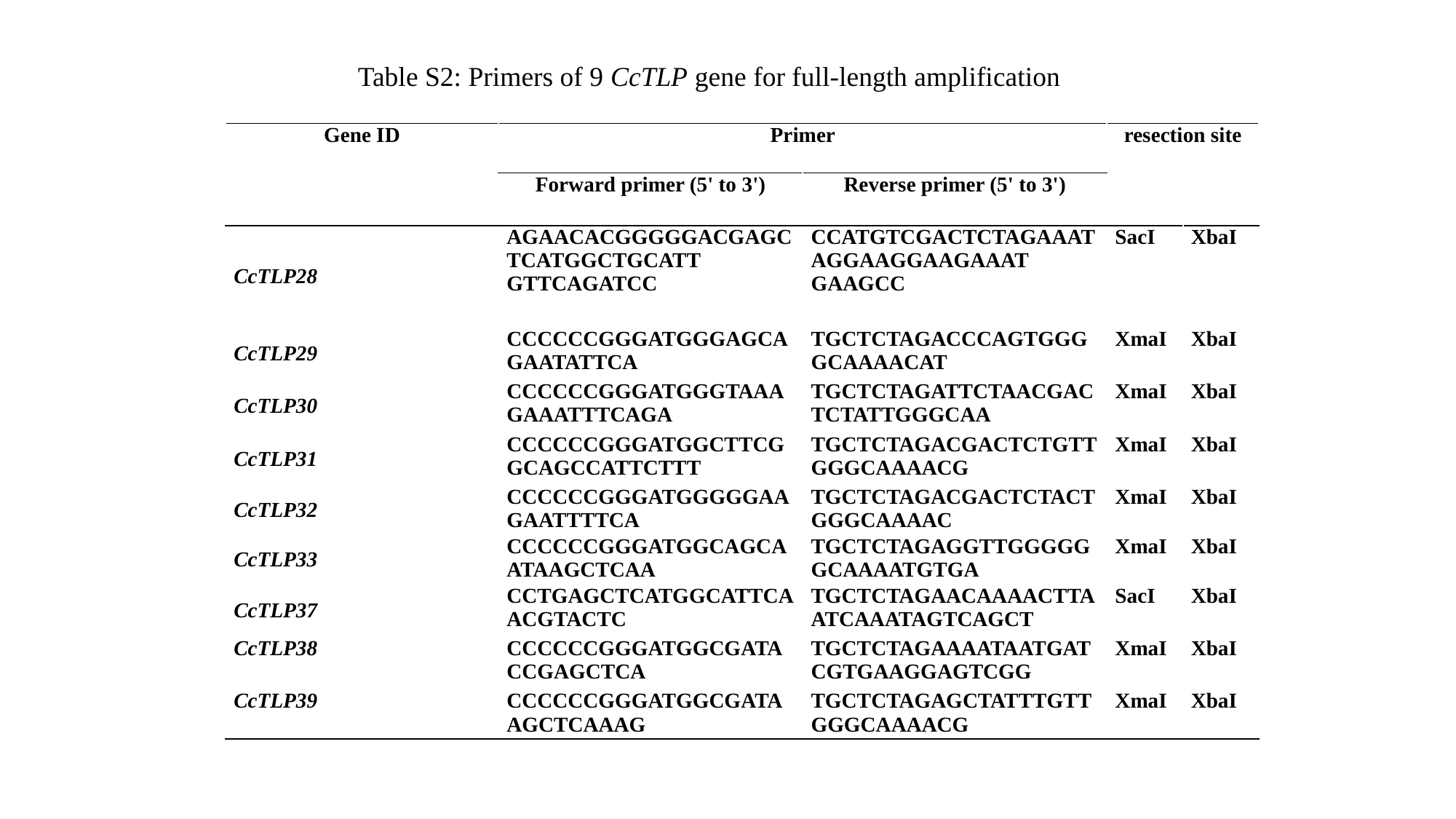

Table S2: Primers of 9 CcTLP gene for full-length amplification
| Gene ID | Primer | | resection site | |
| --- | --- | --- | --- | --- |
| | Forward primer (5' to 3') | Reverse primer (5' to 3') | | |
| CcTLP28 | AGAACACGGGGGACGAGCTCATGGCTGCATT GTTCAGATCC | CCATGTCGACTCTAGAAATAGGAAGGAAGAAAT GAAGCC | SacI | XbaI |
| CcTLP29 | CCCCCCGGGATGGGAGCAGAATATTCA | TGCTCTAGACCCAGTGGGGCAAAACAT | XmaI | XbaI |
| CcTLP30 | CCCCCCGGGATGGGTAAAGAAATTTCAGA | TGCTCTAGATTCTAACGACTCTATTGGGCAA | XmaI | XbaI |
| CcTLP31 | CCCCCCGGGATGGCTTCGGCAGCCATTCTTT | TGCTCTAGACGACTCTGTTGGGCAAAACG | XmaI | XbaI |
| CcTLP32 | CCCCCCGGGATGGGGGAAGAATTTTCA | TGCTCTAGACGACTCTACTGGGCAAAAC | XmaI | XbaI |
| CcTLP33 | CCCCCCGGGATGGCAGCAATAAGCTCAA | TGCTCTAGAGGTTGGGGGGCAAAATGTGA | XmaI | XbaI |
| CcTLP37 | CCTGAGCTCATGGCATTCAACGTACTC | TGCTCTAGAACAAAACTTAATCAAATAGTCAGCT | SacI | XbaI |
| CcTLP38 | CCCCCCGGGATGGCGATACCGAGCTCA | TGCTCTAGAAAATAATGATCGTGAAGGAGTCGG | XmaI | XbaI |
| CcTLP39 | CCCCCCGGGATGGCGATAAGCTCAAAG | TGCTCTAGAGCTATTTGTTGGGCAAAACG | XmaI | XbaI |

## Slide 3
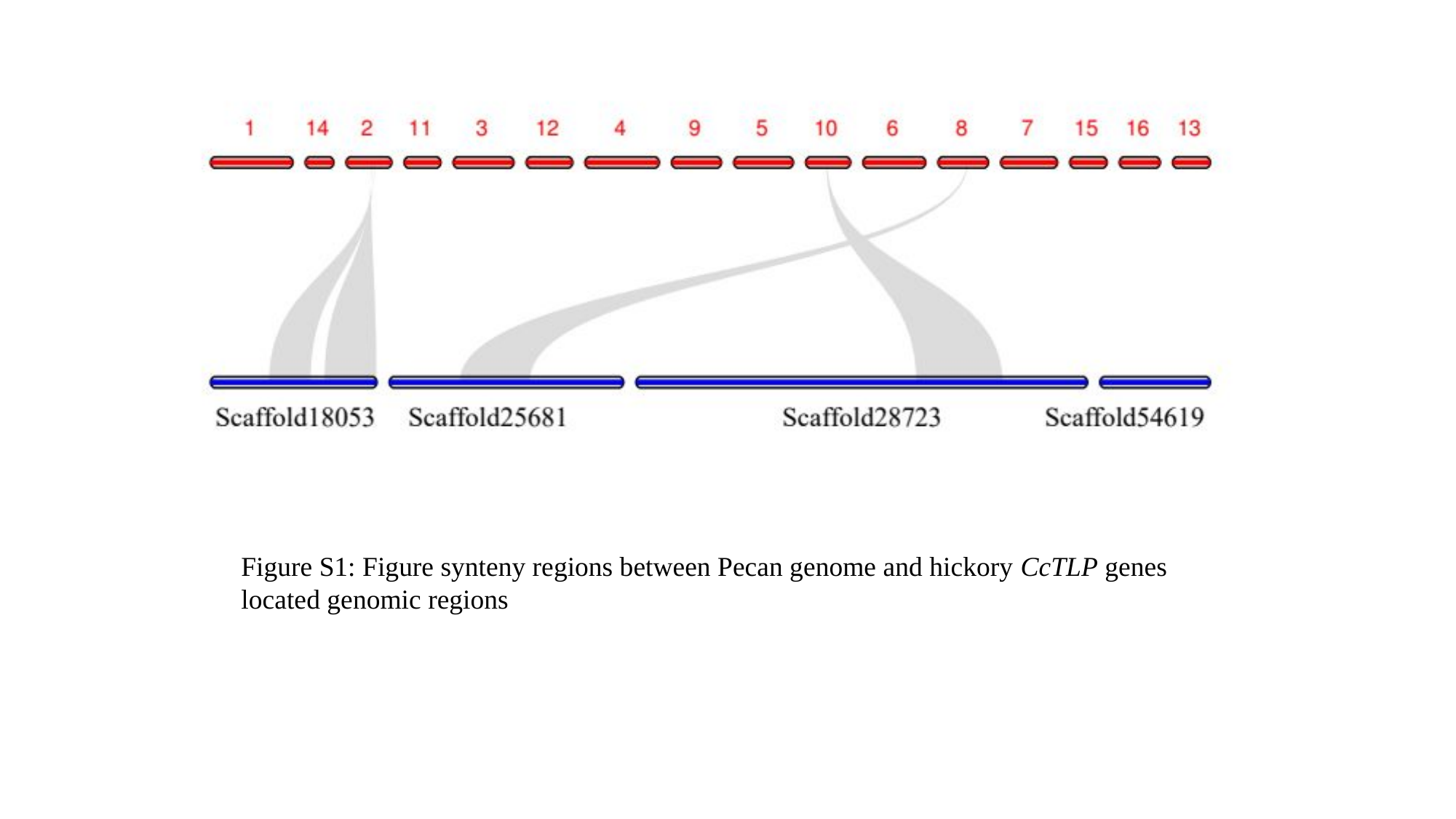

Figure S1: Figure synteny regions between Pecan genome and hickory CcTLP genes located genomic regions

## Slide 4
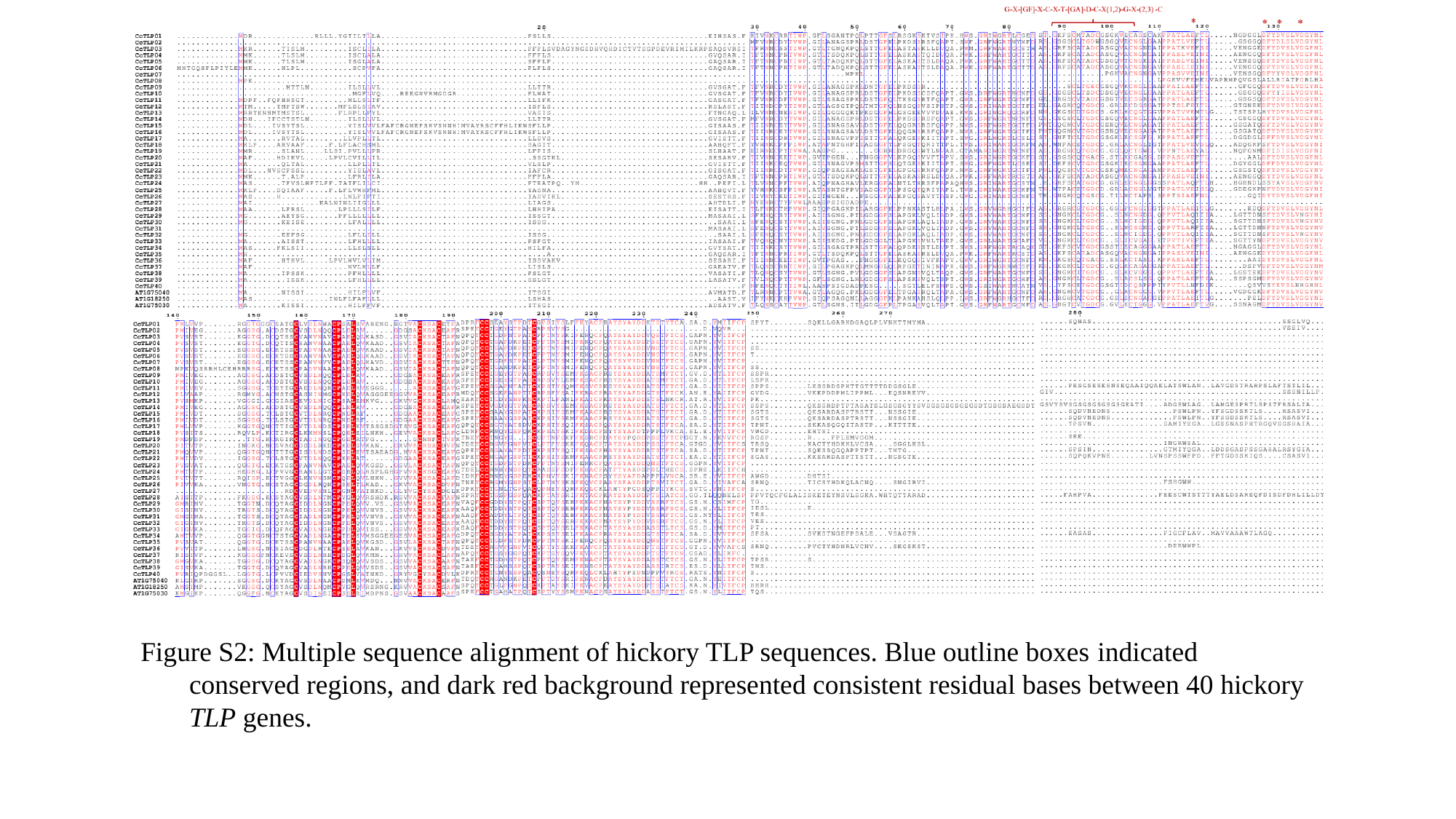

Figure S2: Multiple sequence alignment of hickory TLP sequences. Blue outline boxes indicated conserved regions, and dark red background represented consistent residual bases between 40 hickory TLP genes.

## Slide 5
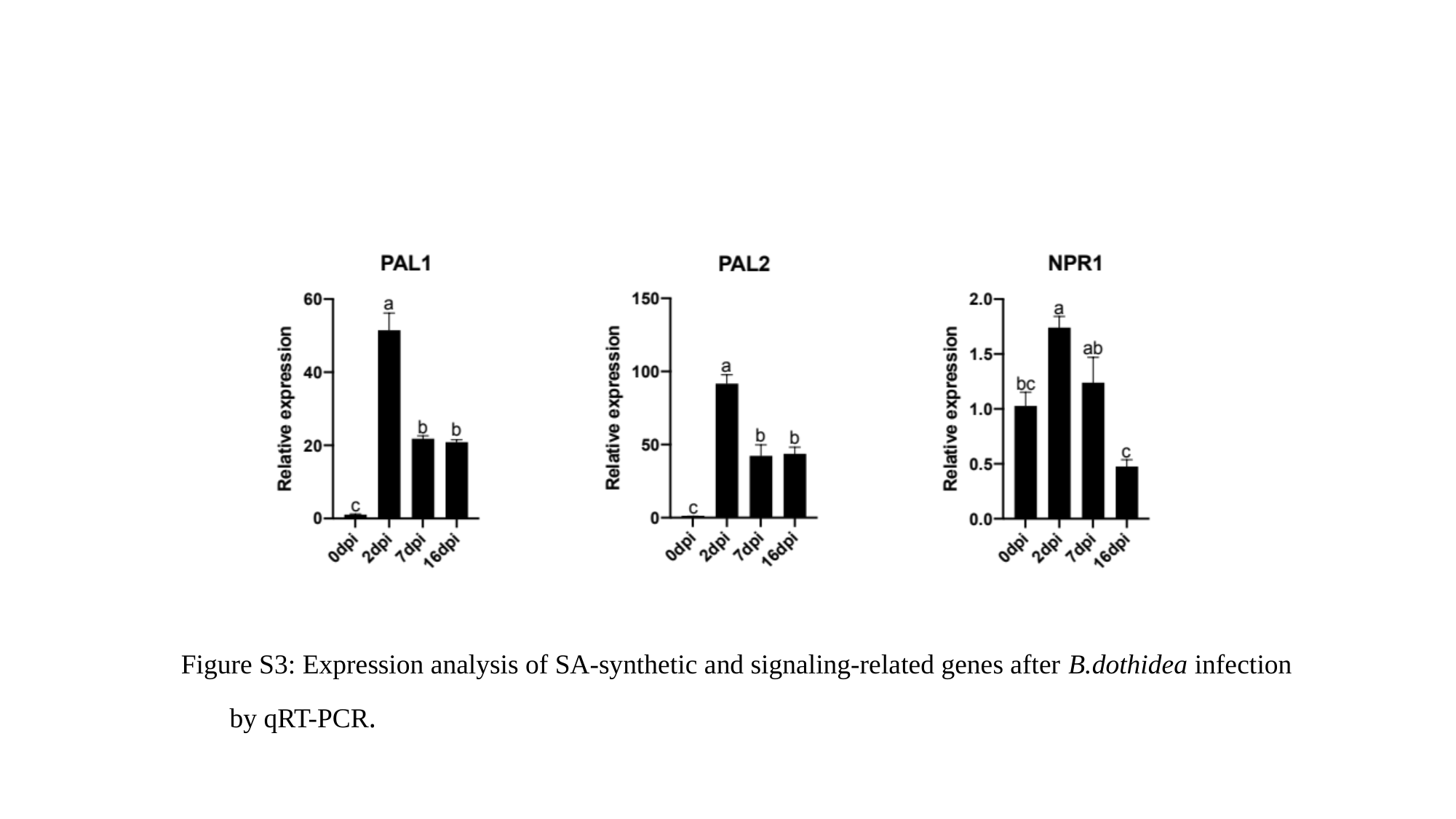

Figure S3: Expression analysis of SA-synthetic and signaling-related genes after B.dothidea infection by qRT-PCR.
